# Spinal Intrathecal Actinomycosis Causes Multisegmental Root Failure: A Case Report

**DOI:** 10.3389/fneur.2020.00621

**Published:** 2020-06-30

**Authors:** Yanying Wang, Xinyu Ren, Dongchao Shen, Chenhui Mao, Han Wang, Bin Peng, Jun Gao, Liying Cui

**Affiliations:** ^1^Department of Neurology, Peking Union Medical College Hospital, Chinese Academy of Medical Sciences, Peking Union Medical College, Beijing, China; ^2^Department of Pathology, Peking Union Medical College Hospital, Chinese Academy of Medical Sciences, Peking Union Medical College, Beijing, China; ^3^Department of Neurosurgery, Peking Union Medical College Hospital, Chinese Academy of Medical Sciences, Peking Union Medical College, Beijing, China; ^4^Neurosciences Center, Chinese Academy of Medical Sciences, Beijing, China

**Keywords:** actinomycosis, intraspinal infection, nerve roots, compression, histopathology

## Abstract

Actinomycosis is a slowly progressing infection caused by *Actinomyces* species, which consists of filamentous gram-positive bacteria. Intraspinal actinomycosis is very rare and most of the previous cases presented with epidural lesions. Only two cases of intrathecal actinomycosis have been described. We reported a case of intrathecal actinomycosis in a 46-year-old woman. Our patient presented with multisegmental root failure, which was different from previous intrathecal cases mainly involving the spinal cord. The manifestations, cervical MR imaging results, biopsy and histopathological features, and treatment history of the patient were reviewed. Clinical features of this disease resemble intraspinal neoplasms, other infectious processes, and granulomatous diseases, thus being difficult to diagnose preoperatively. Histopathological evidence from the biopsy is important for timely diagnosis. Early diagnosis and treatment may greatly improve the prognosis.

## Background

Actinomycosis is a chronic infection caused by *Actinomyces* species, which consists of gram-positive bacteria and members of endogenous flora in the oral cavity, the gastrointestinal tract, and the vagina (1). *Actinomyces* species becomes pathogenic if the integrity of the mucosal barrier is compromised (1).

Actinomycosis is divided into cervicofacial (55%), abdominopelvic (20%), thoracic (15%), and other types (10%) (2). Central nervous system (CNS) actinomycosis is a rare entity, and may manifest as brain abscess, meningitis or meningoencephalitis, actinomycoma, subdural empyema, and epidural abscess (3). Most of the previous cases of intraspinal actinomycosis involved patients who presented with epidural mass lesions (4, 5). For spinal “subdural” lesions, the term “intrathecal” instead of “subdural” is preferred because the latter limits the location to extra-arachnoid (6). Spinal intrathecal actinomycosis is extremely rare and only two cases have been published (5, 6). Here, we present a case of intrathecal actinomycosis mainly involving multisegmental root failure without clinical manifestations of myelopathy. This clinical feature has not been previously reported, to our knowledge, and could help further understand this disease.

## Case Presentation

A 46-year-old female functionary presented to the department of neurology in our hospital with progressive left arm pain and weakness for 3 months. The excruciating radiating pain in her left shoulder and arm occurred 3–4 times every hour and lasted for 10 min per episode. Sustained weakness of the left arm made her unable to comb her hair. She denied fever before or during the disease, but she lost 2 kg of weight because of poor appetite due to the pain. The patient had a history of meningioma resection 3 years ago. However, she denied any intracranial symptoms, and the surgical incision healed well. Recent reexamination of brain MRI was also normal. There was no history of trauma or dental procedures. The patient was allergic to amoxicillin.

Upon physical examination, the patient was afebrile with normal vital signs. No lymphadenopathy was palpated. Cardiovascular, respiratory and abdominal examinations were unremarkable. Upon neurologic examination, the cranial nerve examination was normal. Weakness and atrophy of the following muscles were noted: deltoid (Medical Research Council [MRC] grade 4 -/5), triceps (MRC 3/5), biceps (MRC 3/5), and distal muscles (MRC 4/5) of the left upper limb. The muscle tone of the left upper limb was slightly decreased. All tendon reflexes were reduced in the left upper limb. Sensory examination revealed hypoalgesia on the lateral side of the left upper limb, left thumb, and index finger. Pathological reflexes and meningeal irritation were negative.

**Figure 1:**
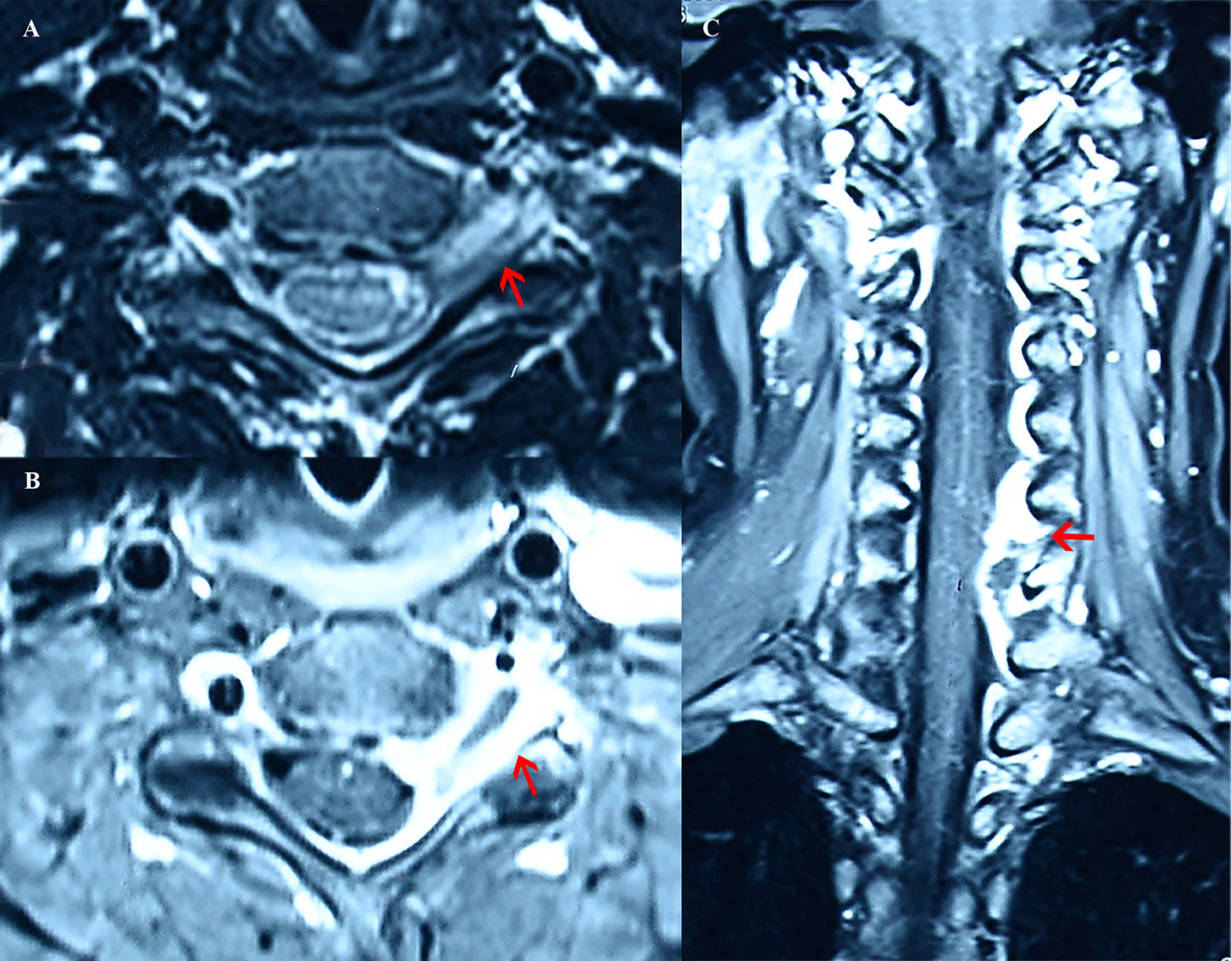
Cervical MRI images from the patient. **(A)** On axial MRI, at the C5–C6 level, the lesion (the red arrow) partially surrounded the left vertebral artery and extended through the left intervertebral foramen into the spinal canal, with T2 mixed intensity. **(B)** On axial MRI, at the C5-C6 level, the lesion (the red arrow) exhibited gadolinium enhancement around and T1 hypointensity in the center. **(C)** On coronal MRI, the lesion spread from C4 to C7 in the spinal canal, especially demonstrating mass effect (the red arrow) at the C5–C6 level. The lesion showed uneven gadolinium enhancement with T1 hypointensity in the center.

The patient had previously undergone cervical spine magnetic resonance imaging (MRI) elsewhere. At the C5–C6 level, the lesion partially surrounded the left vertebral artery and extended through the left intervertebral foramen into the spinal canal (Figures 1A,B). On coronal MRI, the lesion spread from C4 to C7 in the spinal canal, especially demonstrating mass effect at the C5–C6 level (Figure 1C).

The electromyogram only revealed spontaneous potential in the left biceps brachii. Motor conduction studies of the bilateral median and ulnar nerves showed normal findings, as did the somatosensory evoked potential of the left upper limb.

Laboratory tests showed normal hemoglobin levels, total leucocyte count, platelet count, and erythrocyte sedimentation rate. T-SPOT.TB was positive, but a further Xpert MTB/RIF test of the sputum was negative. The following results were all normal: serologic tests for syphilis, Lyme disease, and HIV; serum autoimmune screening; and angiotensin-converting enzyme level measurements. A whole-body CT scan did not provide any evidence of underlying malignancy.

Upon lumbar puncture, the opening pressure was normal, and the cerebrospinal fluid (CSF) was clear and colorless. The CSF white blood cell count was elevated (505/mm^3^); lymphocytes (50%) and neutrophils (40%) were predominant. The CSF protein level was 70 mg/dL, and the CSF glucose level was 41.4 mg/dL. The CSF bacterial smear, bacterial culture, acid-fast staining, and fungal smear results were negative. No abnormalities were found in the CSF anti-GM1 antibody test. CSF cytology was negative for malignancy.

The patient's clinical manifestations indicated C5–C7 radiculopathy. The MRI findings confirmed the location of C4–C7 myeloradiculopathy. Combining the CSF profile of leukocytic pleocytosis, high protein level, and hypoglycorrhachia, inflammatory lesions were considered most likely. The patient was empirically treated with 2 g of intravenous ceftriaxone daily and with pulse intravenous methylprednisolone.

**Figure 2:**
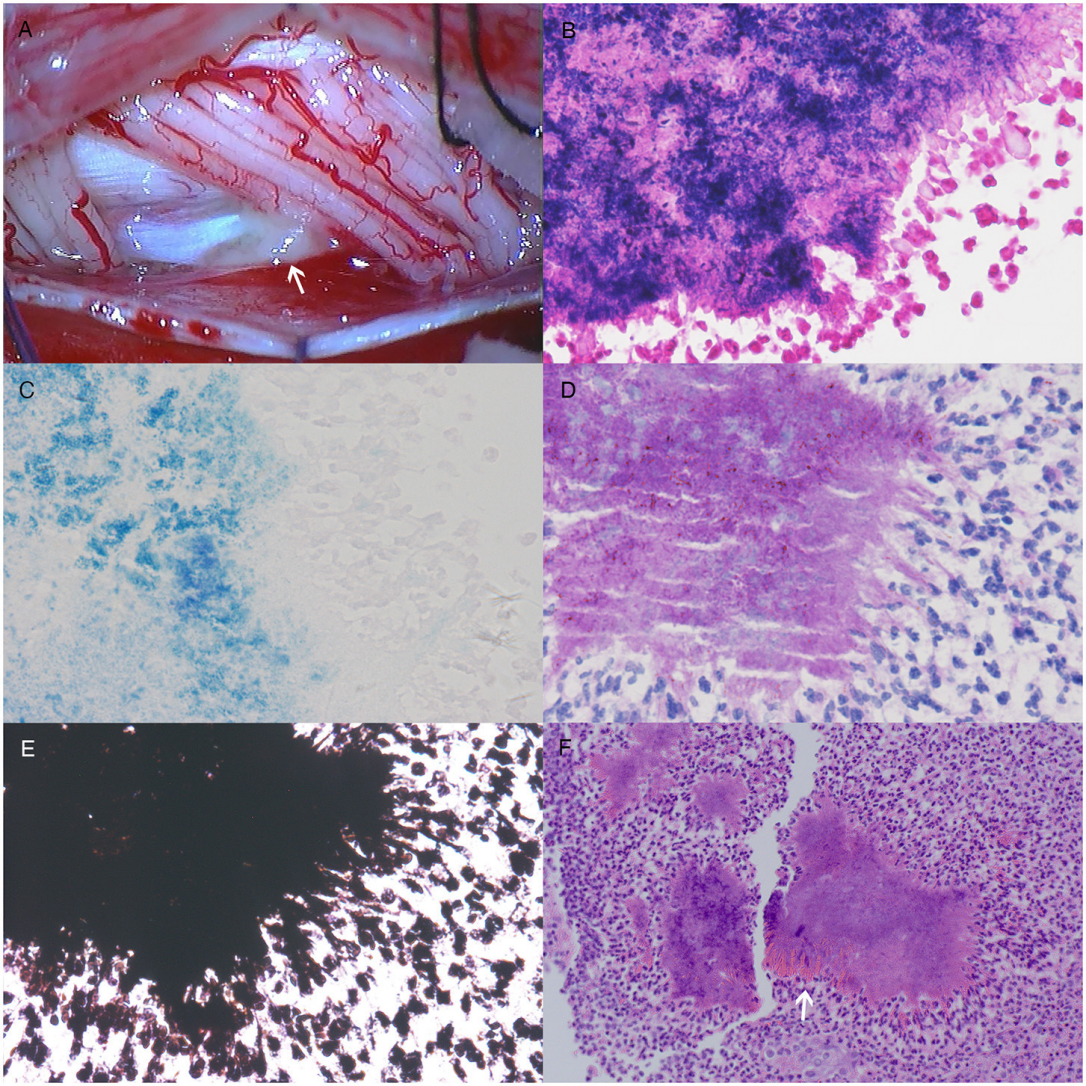
The photograph and histological staining of the intraspinal biopsy. **(A)** During the biopsy, an intrathecal gray-white mass on the left ventral side of the C5–C6 spinal cord surrounding the left C5–C6 nerve roots was observed. The mass had a brittle texture and rich blood supply, and the base of the mass was on the dura mater. Histological staining shows a colony of Gram-positive (×400) **(B)**, acid-fast staining negative (×400) **(C)**, periodic acid-Schiff staining positive (×400) **(D)**, and Grocott's methenamine silver staining positive (×400) **(E)** filamentous branching bacteria, with hematoxylin and eosin staining revealing the Splendore-Hoeppli phenomenon (×200, the white arrow on **F**). A chronic inflammatory process around the bacteria colony was also observed (×200, **F**).

To establish the final diagnosis, an intraspinal biopsy was performed. The dural sac was opened from C5 to C6. During the biopsy, we observed an intrathecal gray-white mass on the left ventral side of the C5–C6 spinal cord surrounding the left C5–C6 nerve roots. The mass had a brittle texture and rich blood supply, and the base of the mass was on the dura mater (Figure 2A). The mass was partially resected, and we did not further explore the intervertebral foramen and epidural lesions to avoid bleeding and the spread of potential infection. Histopathological examination of the resected mass showed a colony of Gram-positive, acid-fast staining negative, periodic acid-Schiff staining positive and Grocott's methenamine silver staining positive filamentous branching bacteria, with hematoxylin and eosin (HE) staining revealing the Splendore-Hoeppli phenomenon (Figures 2B–F). In addition, histopathological examination also indicated a chronic inflammatory process involving epidural structures. Therefore, the patient was diagnosed with intraspinal actinomycosis based on the pathological evidence. However, no definite pathogenic microorganism was identified with tissue culture.

The intravenous dosage of ceftriaxone was then increased to 2 g twice daily, and prednisone acetate sequential therapy was administered. After a 3-month treatment regimen, the patient showed favorable neurological progression. Her left arm pain disappeared, and the strength of the proximal muscles increased to MRC 4+/5 and that of the distal muscles to MRC 5/5. The CSF white blood cell count decreased to 22/mm^3^.

## Discussion

Actinomycosis, caused by *Actinomyces* species, was first described by Israel in 1878 (7). Intraspinal actinomycosis is a very rare disease, and only 29 cases have been reported so far (8, 9). The majority of patients with intraspinal actinomycosis are immunocompetent (8). The disease tends to affect adults, and a male predominance is observed (8). The dura surrounding the spinal cord is a tight barrier that prefers epidural abscess and granulation tissue formation rather than intrathecal empyema (10). Therefore, intraspinal actinomycosis is characterized by epidural mass lesions, while only two cases of intrathecal actinomycosis have been reported (5, 6).

The first reported case of intrathecal actinomycosis was thought to result from dissemination of a preexisting intracranial abscess (6). However, in the second reported case, no preexisting source of infection was found (5). In our case, combining imagining, biopsy and pathological findings, we speculated that the infection might primarily locate epidurally, and spread through the relatively weak nerve root sleeve at intervertebral foramen, finally resulting in intrathecal infection. However, we only found the intrathecal bacterial colony, and the source that the infection disseminated from was unknown. The lungs were the most common initial infected sites of intraspinal actinomycosis, while in more than half of the cases, no infection source was detected (8). Intracranial actinomycosis with undetected sources more often occurred after an unrelated cranial intervention (11). In this intraspinal actinomycosis case, the patient had a history of meningioma resection, suggesting a potential association with the infection. However, no evidence was found to support this cranial intervention as a potential source. Other risk factors of CNS actinomycosis, such as chronic sinusitis/mastoiditis/gingivitis and dental procedures, congenital heart diseases, infected intrauterine devices, and alcoholism, have also been described (12).

Intraspinal actinomycosis presents with non-specific manifestations and has an insidious onset. Persistent back pain is usually the initial complaint, and only 65% of patients had a history of fever (8). Local neurological symptoms can indicate involvement of the spinal cord or the nerve root segments. The mechanism contributing to myelopathy or myeloradiculopathy may be compression by intraspinal mass lesions, together with inflammation (4). Epidural inflammation may compromise circulation of the epidural venous plexus, resulting in ischemia of the spinal cord and nerve roots (13). Similarly, intrathecal inflammation may lead to arachnoid adhesions, thus compressing the pial venous plexus. Limb weakness, paresis or restricted movement, and hypoesthesia or paresthesia are the most common symptoms (8). These features resemble what is seen with neoplasms, other infectious processes, and granulomatous diseases, making the diagnosis difficult preoperatively, especially when no preexisting focus of infection is documented (8, 10). Our patient presented with multisegmental root failure, which was different from previous intrathecal cases mainly involving the spinal cord (5, 6).

The diagnosis of actinomycosis is made by culture of the pathogen or histopathology. Due to the difficulty of *Actinomyces* cultivation, the diagnosis of intraspinal actinomycosis is primarily based on histopathological evidence from the biopsy (8). Despite the risk of invasive surgery, biopsy remains the recommended method when intraspinal actinomycosis is highly suspected (8). Typical microscopic findings include filamentous Gram-positive fungal-like bacteria and necrosis with yellowish sulfur granules (14). However, sulfur granules comprise <1% of the total tissue and are also present in other infections, including nocardiosis, which resembles *Actinomyces* species in morphological features of Gram staining (15). Further, the Splendore-Hoeppli phenomenon can also be found in the infection with *Nocardia* species (16). The negative result of acid-fast staining can help with differential diagnosis from *Nocardia* species, which exhibits varying degrees of acid fastness (1).

Standards for antimicrobial treatment of intraspinal actinomycosis have not yet been established. Intravenous administration of high doses of penicillin G or amoxicillin for 4–8 weeks is preferred, and continued treatment of oral antibiotics for 6–12 months is usually recommended to prevent relapses and local complications (9, 10). Nonetheless, the complete resolution of symptoms has been described in only 50% of the reported cases (17). Acceptable alternative regimens for intraspinal actinomycosis include tetracycline, erythromycin, and clindamycin (8). *Actinomyces* are almost uniformly susceptible to beta-lactam antibiotics, and ceftriaxone has also been used for central nervous system actinomycosis (14).

## Conclusion

Spinal intrathecal actinomycosis is an extremely rare disease. It presents with non-specific manifestations that resemble intraspinal neoplasms, other infectious processes, and granulomatous diseases, thus being difficult to diagnose preoperatively. Histopathological evidence from the biopsy is important for timely diagnosis. Early diagnosis and treatment may greatly improve the prognosis, thus avoiding severe neurological deficits.

## Data Availability Statement

The datasets generated for this study are available on request to the corresponding author.

## Ethics Statement

This study was reviewed and approved by the Ethics Committee of Peking Union Medical College Hospital. The patients/participants provided their written informed consent to participate in this study.

## Patient Consent

We received written informed consent from the patient for the publication.

## Author Contributions

YW: clinical data review, literature review, and writing the first draft. XR: providing the final pathologic diagnosis and writing the first draft. DS: clinical data review and critical revision of the manuscript for intellectual content. CM, HW, and BP: analyzing the data and intellectual contribution on clinical decision-making. JG: performing the intraspinal biopsy and critical revision of the manuscript for intellectual content. LC: interpreting the data and critical revision of the manuscript for intellectual content.

## Conflict of Interest

The authors declare that the research was conducted in the absence of any commercial or financial relationships that could be construed as a potential conflict of interest.
